# Integrated Glyco-Analytical Strategy for Comprehensive Characterization of a Complex Therapeutic Glycoprotein: Fabrazyme

**DOI:** 10.3390/ijms27083358

**Published:** 2026-04-08

**Authors:** Mikhail Afonin, Polina Novikova, Andrei Vinalev, Natalia Mesonzhnik

**Affiliations:** 1 Resource Centre of Mass-Spectrometry, Laboratory Complex, Sirius University of Science and Technology, Olympic Ave. 1, 354340 Sochi, Russia; afonin.mb@talantiuspeh.ru (M.A.); repin.polina@gmail.com (P.N.); 2 Resource Centre of Analytical Method, Laboratory Complex, Sirius University of Science and Technology, Olympic Ave. 1, 354340 Sochi, Russia; vinalev.aa@talantiuspeh.ru; 3 Center for Genetics and Life Sciences, Sirius University of Science and Technology, Olympic Ave. 1, 354340 Sochi, Russia

**Keywords:** agalsidase beta, Fabrazyme, glycoprotein therapeutics, N-glycosylation, site-specific glycosylation, glycopeptide mapping, intact protein mass spectrometry, released glycan analysis

## Abstract

Fabrazyme (agalsidase beta) is a therapeutic enzyme whose clinical efficacy is contingent upon its complex N-glycosylation patterns. Nevertheless, comprehensive glycosylation profiling remains challenging due to high site-specific heterogeneity. To address this, three orthogonal liquid chromatography–mass spectrometry (LC-MS) approaches were employed: (1) released N-glycan analysis with fluorescence detection and MS annotation, (2) site-specific glycopeptide mapping, and (3) intact protein MS. The released glycan profiling method was validated for reproducibility, intermediate precision, and inter-laboratory transferability, thereby enabling reliable separation and quantification of neutral, phosphorylated, and sialylated species. Glycopeptide mapping revealed distinct site-specific distributions: N108 was found to predominantly carry sialylated complex glycans; N161 was enriched in phosphorylated oligomannose structures; and N184 displayed the highest heterogeneity, including bisphosphorylated and sialylated glycans. Intact protein analysis was performed on both intact and desialylated Fabrazyme, thereby enabling confirmation of glycan assignments. Desialylation reduced spectral complexity, thereby facilitating accurate mass matching with a combinatorial library generated from glycopeptide-level data. The complementary use of these three analytical levels provides a comprehensive view of Fabrazyme glycosylation, offering a robust reference for quality control and biosimilar development.

## 1. Introduction

Fabry disease is a rare X-linked lysosomal storage disorder that results from a deficiency of the α-galactosidase A (α-Gal A) enzyme [1,2,3]. The enzyme deficiency leads to the accumulation of glycosphingolipids, mainly globotriaosylceramide (Gb3), in multiple cell types, resulting in severe clinical manifestations [3,4].

The treatment regimen comprises supportive care and timely administration of enzyme replacement therapy (ERT) using either agalsidase alfa (Replagal), produced in a continuous human cell line [5], or agalsidase beta (Fabrazyme), produced in Chinese hamster ovary cells using recombinant DNA technology [6,7,8]. Both agalsidases catalyse the hydrolysis of Gb3 and other glycosphingolipids to ceramide dihexoside and galactose. Following intravenous administration, the enzyme is rapidly cleared from the circulation; its uptake by vascular endothelial and parenchymal cells into lysosomes is mediated by the mannose-6-phosphate receptor (M6PR) and other receptors [5,6,8,9].

It has been reported that there are variations in the clinical outcomes of both ERT treatments, which are likely influenced by a number of factors, including the timing of therapy, disease phenotype, and the extent of irreversible damage [10,11,12,13,14,15]. Notably, high-dose agalsidase beta exhibits slightly greater effectiveness than agalsidase alfa regarding both cardiovascular and renal events [10,11,12,16].

Both proteins share an identical amino acid (AA) sequence with the native human enzyme but differ in their glycosylation patterns, which are determined by the cell line used for production. Agalsidase beta is a non-covalently linked homodimer with an approximate molecular weight (MW) of 100 kDa. Each monomer, with a theoretical mass of 45,349 Da, consists of 398 AA residues and is N-glycosylated at asparagines 108, 161, and 184 (Figure S1). These sites predominantly host distinct glycan structures: fucosylated biantennary bisialylated complex, monophosphorylated mannose oligomannose, and biphosphorylated mannose oligomannose, respectively [5,8,9].

The therapeutic efficacy and stability of agalsidase-based therapeutics are strongly influenced by their N-glycosylation patterns. This well-established post-translational modification (PTM) is critical for protein folding, stability, and function. Additionally, glycosylation impacts the pharmacokinetics, immunogenicity, and bioactivity of therapeutic proteins. In particular, the N-linked glycans present at asparagine residues N161 and N184 predominantly host oligomannose-type glycans, which are unique in containing the lysosomal targeting signal, M6P. The M6P modification enables the enzyme to bind to mannose-6-phosphate receptors (M6PR) on cell surfaces, thereby facilitating its uptake into cells and delivery to lysosomes, where it performs the hydrolysis of Gb3. It has been demonstrated that mutations or alterations in glycosylation at these sites can impair the trafficking of the enzyme, resulting in a reduction in its therapeutic effectiveness [17].

Despite the clinical importance of Fabrazyme, comprehensive glycosylation profiling studies that integrate multiple orthogonal analytical techniques remain scarce. Previous studies have addressed individual aspects of its glycosylation; however, a systematic investigation integrating glycan characterisation across multiple analytical levels has not been reported. Such an integrated approach is essential to fully capture the structural heterogeneity that may impact product quality, batch consistency, and biosimilar development.

In this study, we examine the applicability of an integrated multilevel LC-MS strategy for comprehensive characterisation of the complex N-glycosylation pattern of Fabrazyme. By leveraging high-resolution mass spectrometry (HRMS), we systematically characterised released glycans, glycopeptides, and intact protein glycoforms.

The RapiFluor-MS labeled released glycan analysis, a conventional approach for glycoprofiling, was optimised to improve the chromatography resolution of glycan species and maximize their recovery. The method was validated, thereby establishing it as a robust tool for routine quality control.

Glycopeptide mapping revealed a broad repertoire of glycan species distributed across the three glycosylation sites, with site-specific specialisation that would be inaccessible by conventional released glycan analysis alone.

In order to reduce spectral complexity at the intact protein MS level and facilitate glycoform annotation, we employed enzymatic desialylation, which enabled correlation between desialylated and sialylated proteoforms. The wealth of molecular information obtained at lower structural levels was integrated to support the interpretation of intact mass spectra through the application of a combinatorial library generated from glycopeptide data.

The methodologies described herein establish a comprehensive framework for Fabrazyme glycosylation analysis that has not previously been available, and can be utilised for batch-to-batch consistency assessment and biosimilar comparability studies.

## 2. Results

### 2.1. Released Glycan Profiling by LC-FLD with MS Annotation

The analysis of released N-glycans from glycoproteins poses significant analytical challenges, primarily because phosphorylated glycan species are prone to adsorptive losses on surfaces encountered during sample preparation (e.g., plasticware and stationary phases). To address this issue, a modified version of the manufacturer’s GlycoWorks™ (Waters, Milford, MA, USA) protocol was applied to the analysis of Fabrazyme. This protocol incorporates RapiGest SF surfactant, PNGase F, and the RapiFluor-MS labeling reagent. The conventional protocol was modified by the incorporation of a dedicated wash with sodium citrate-containing buffer and an additional ACN-based purification step. Sodium citrate acts as a chelating agent, mitigating the adsorptive loss of sialylated and phosphorylated glycans—particularly bisphosphorylated species—during hydrophilic interaction liquid chromatography (HILIC) sample clean-up. The implementation of an additional purification step was imperative for the effective removal of buffer components, excess RapiFluor-MS reagent, and unreacted by-products [18].

A critical aspect of the analytical strategy was the choice of chromatographic column. Conventional amide HILIC stationary phases typically result in co-elution of phosphorylated glycans with other glycan species. To overcome this limitation, a mixed-mode C18 anion exchange (MMAX) column was employed, which enabled superior resolution of phosphorylated glycans from neutral and sialylated species, thereby allowing reliable quantification.

**Figure 1:**
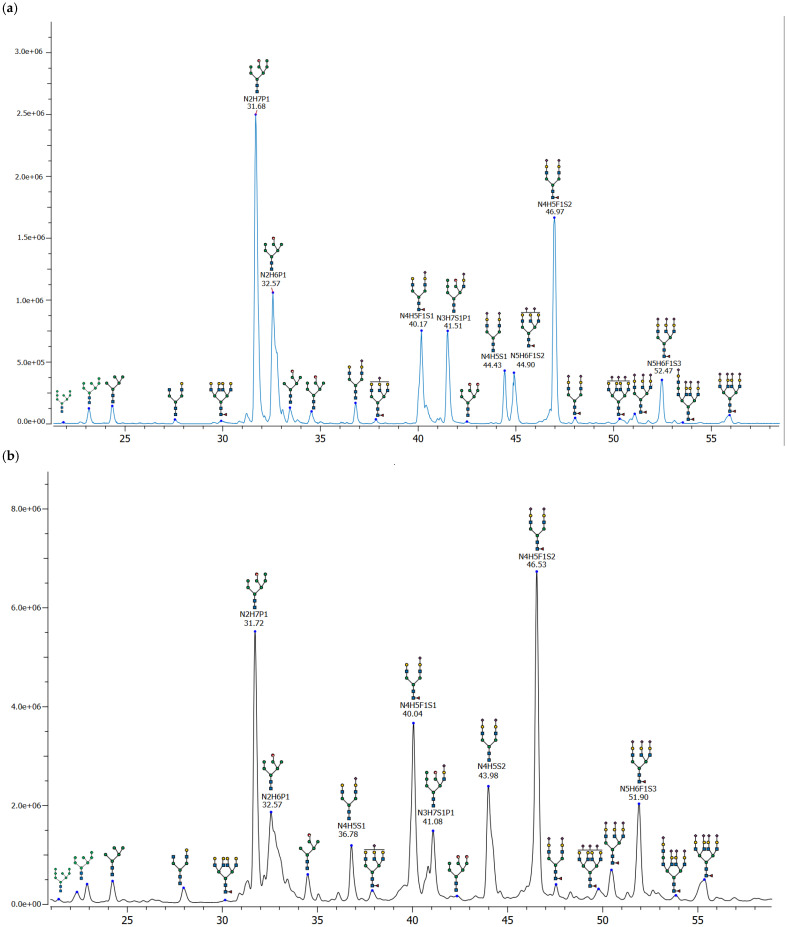
Released RapiFluor N-glycan profiling of Fabrazyme. The identified glycan species are annotated with their proposed structures: (**a**) base peak intensity chromatogram obtained by LC-MS analysis and (**b**) Fluorescence chromatogram (λex = 265 nm, λem = 425 nm).

Because the FLD and MS detectors were operated on separate UPLC systems, chromatographic traces were aligned on the basis of retention time using reference peaks to permit direct comparison of the glycan profiles. The aligned FLD and MS profiles are presented in Figure 1a and Figure 1b, respectively, with major glycan species indicated. The LC-FLD chromatogram obtained using the MMAX column provided excellent resolution of the released glycan species. Peak annotation, performed using MS data, demonstrated the high resolving power of the method, enabling clear separation of phosphorylated glycans from neutral and sialylated species.

Characterization of Fabrazyme N-glycans by LC-FLD with MS annotation demonstrated substantial structural heterogeneity, including oligomannose, hybrid, and complex-type species.

The chromatographic separation of the MMAX column enabled the distinct resolution of glycan classes based on their structural features. Phosphorylated oligomannose glycans were eluted during the initial phase of the chromatographic analysis, with the retention times of these glycans demonstrating a direct correlation with the number of mannose residues present. The most abundant among these were the monophosphorylated species N2H7P1 and N2H6P1, while bisphosphorylated variants (e.g., N2H7P2, N2H6P2) were present at lower abundances. A phosphorylated hybrid structure, N3H7S1P1, containing both a phosphate and a sialic acid residue, was also detected as a notable component.

Sialylated glycans were predominant in the later-eluting region of the chromatogram, with separation driven by the degree of sialylation and antennarity. Biantennary fucosylated monosialylated (N4H5F1S1) and disialylated (N4H5F1S2) glycans were the most abundant species within this class. Triantennary and tetraantennary structures (e.g., N5H6F1S2, N5H6F1S3, N6H7F1S3, N6H7F1S4) were also observed as reproducible minor components, further contributing to the overall microheterogeneity.

In total, 51 chromatographic peaks corresponding to 43 distinct N-glycan compositions were identified, including 9 phosphorylated, 21 sialylated, and 14 neutral glycans (of which 7 were high-mannose structures). A comprehensive list of identified glycans is provided in Table S1. The separation performance of this method enabled the reliable identification and relative quantification of all major and minor glycan species, forming the basis for subsequent method validation.

Accordingly, the method was subsequently validated for intra-laboratory repeatability, intermediate precision, and inter-laboratory transfer. Validation results, including statistical parameters (mean, standard deviation (SD), relative standard deviation (RSD), Dixon’s and Anderson–Darling tests), summarized in Tables S2–S5. The definition and classification of glycan groups used for validation are provided in the Supplementary Materials (see note preceding Table S2).

Repeatability assessments (2 analysts, *n* = 6) demonstrated low relative standard deviations (RSD ≤ 1.6% for all groups), with no significant outliers detected (*p* > 0.05) and data normality confirmed (*p* > 0.05). Intermediate precision (*n =* 12) yielded RSD values ranging from 0.2% to 9.9%, with comparable variances confirmed for most groups and no significant bias observed between analysts. Inter-laboratory transfer was successful, as all measurements obtained on the second UPLC system fell within the pre-established ±3σ acceptance limits. Collectively, these validation results confirm that the method is robust, reproducible, and suitable for its intended purpose.

While the released glycan analysis enabled comprehensive profiling of overall N-glycan heterogeneity, this conventional approach provides the identity and relative abundance of individual glycans, but does not reveal their distribution across the glycosylation sites of Fabrazyme. To determine site-specific glycosylation heterogeneity, we next performed glycopeptide mapping of the three N-glycosylation sites.

### 2.2. Glycopeptide Mapping

To assess the site occupancy of Fabrazyme, a comparative evaluation of various glycopeptide separation strategies was undertaken. Glycan profiles were analyzed using two different column chemistries: Waters ACQUITY Premier BEH Peptide C18 (130 Å) and Waters ACQUITY Premier CSH Peptide C18 (130 Å). Furthermore, the impact of an elevated FA concentration in the mobile phases on glycopeptide resolution was investigated specifically for the CSH column. As previously reported in the literature [19], the addition of 0.3% or more FA to both mobile phases during gradient elution on this stationary phase can potentially enhance the separation, improve peak symmetry, and increase the signal response for charged glycopeptide species.

**Figure 2:**
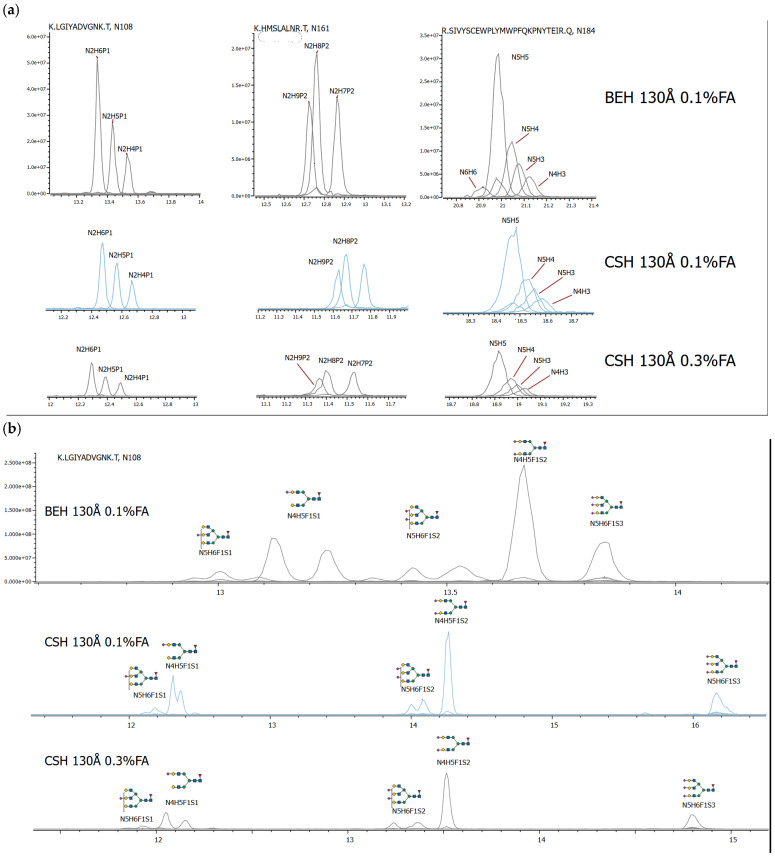
Comparison of Fabrazyme glycopeptide separation under different conditions. (**a**) XIC of phosphomannose, diphosphomannose, and neutral glycopeptides; (**b**) XIC of sialylated glycopeptides. Intensities are shown on the same scale.

Our analysis revealed that, under identical conditions, the BEH Peptide C18 phase provided significantly superior separation quality and sensitivity for neutral and phosphorylated glycopeptides compared to the CSH Peptide C18 phase (Figure 2a). Conversely, for charged, sialylated glycopeptides, the CSH stationary phase demonstrates a clear advantage, resulting in a markedly different separation profile and enabling the differentiation of glycoforms based on their sialic acid content (Figure 2b). The incorporation of 0.3% FA into both mobile phases did not yield a significant improvement in the separation for most glycopeptide types, with the exception of sialylated species, where enhanced resolution between isomeric peaks was observed. However, this elevated acid concentration led to a substantial decrease in detection sensitivity (up to 5-fold) when compared to the performance of the BEH Peptide C18 column under standard conditions.

A glycopeptide mapping analysis was performed using HRMS in order to characterise the glycosylation profile of Fabrazyme. Glycopeptides were separated on an analytical column of a BEH C18 peptide, and then subjected to CID in a DDA mode. This analysis confirmed the presence of three N-linked glycosylation sites located at asparagine residues N108, N161, and N184, which is consistent with the known structure of human α-Gal A [8], and multiple glycan species were identified per site.

Accurate and detailed glycopeptide assignment was performed using Byos software version 5.10 (Protein Metrics Inc., Boston, MA, USA). Representative annotated glycopeptide spectra for each glycosylation site are provided in the Supplementary Materials (Figures S2–S4).

The glycopeptide identifications and relative abundances for the N-glycosylation site of Fabrazyme are presented in the Supplementary Materials (Tables S6–S8).

Site occupancy was determined to be near 100% for N108 and N161. Notably, only the deamidated form of the aglycosyl peptide (N108_agly_dea) was observed at N108 (0.38%). This may reflect enhanced susceptibility of the unoccupied N108 residue to deamidation under the experimental conditions employed. The non-glycosylated peptide form of N161 (N161_agly + N161_dea) accounted for 2.9% of the total peptide response. In contrast, the aglycosyl form of the peptide corresponding to N184 (N184_agly) was observed at 19.5%. While these findings indicate near-complete occupancy at most sites, the elevated aglycosyl fraction at N184 warrants further consideration. This may be indicative of a partial occupancy of the site during post-translational processing in the manufacturing cell line. However, it is also possible that the glycopeptides carrying the phosphorylated and sialylated glycans of N184 exhibit lower ionisation efficiency compared to their aglycosyl counterpart, leading to an overestimation of the unoccupied fraction [20]. Overall, the distribution and structural features of the glycans were found to be highly site-specific.

### 2.3. Glycosylation at N108

**Figure 3:**
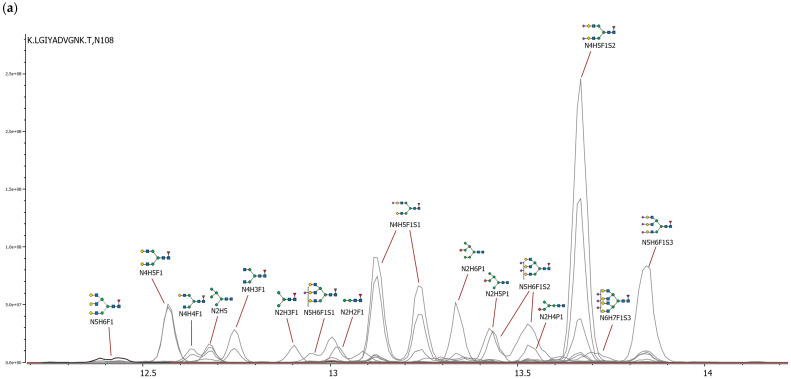

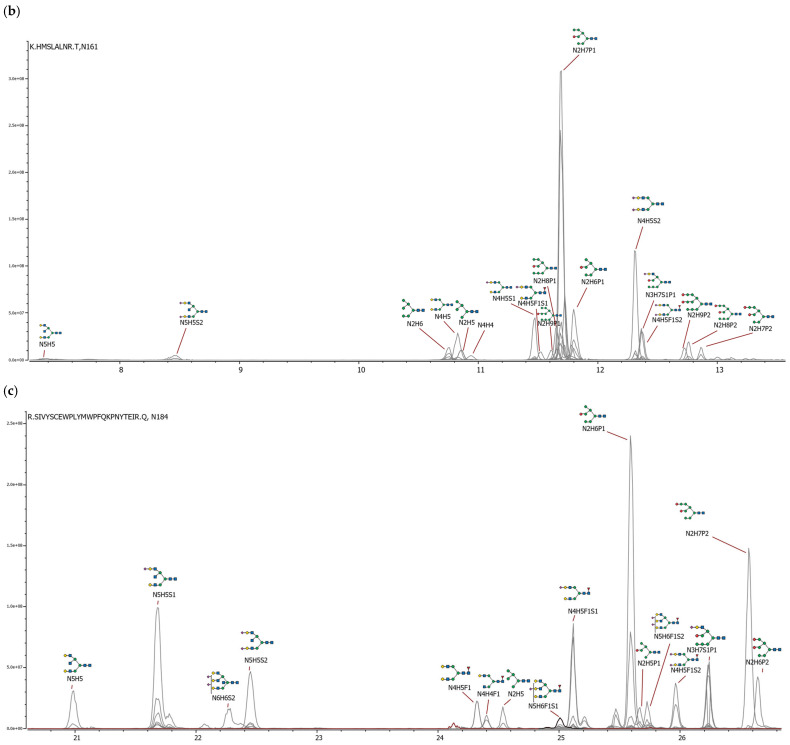
Glycopeptide mapping of the N-glycosylation sites of Fabrazyme. XICs of glycopeptides corresponding to (**a**) N108, (**b**) N161, and (**c**) N184.

A total of 30 distinct glycan species were identified at the N108 site (Figure 3a). The glycan population is dominated by complex-type oligosaccharides, which represent approximately 97.5% of the total glycoforms. The predominant species identified in this study are fucosylated, sialylated bi-, tri-, and tetra-antennary complex structures. The most abundant individual glycan is the disialylated, fucosylated biantennary structure (N4H5F1S2), comprising 27.8% of the total glycans at this site, followed by its monosialylated variant (N4H5F1S1), which accounts for 20.1% when combining both chromatographic peaks of isomers corresponding to this glycan (11.8% and 8.3%).

**Figure 4:**
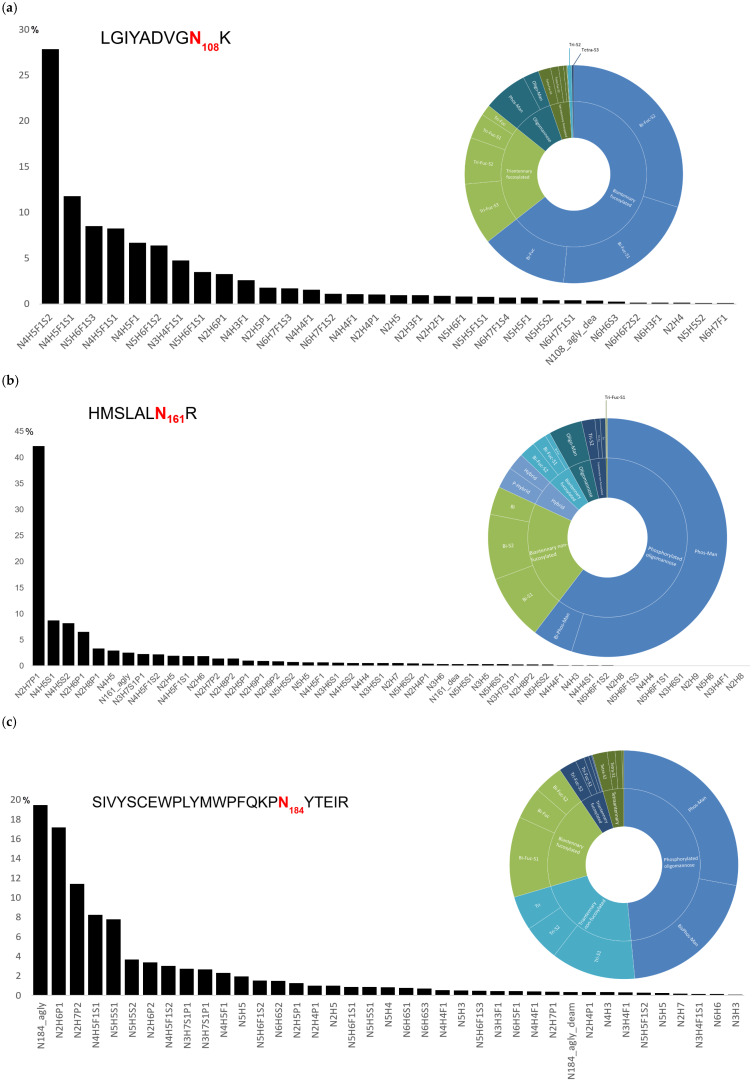
Site-specific glycan distribution across the N-glycosylation sites of Fabrazyme. Relative abundances of glycan classes at (**a**) N108, (**b**) N161, and (**c**) N184. Glycans are grouped by structural features.

Among the complex-type glycans, biantennary structures are the most prevalent, constituting approximately 60% of the total glycoforms at N108 (Figure 4a). This class includes fucosylated disialylated (27.8%), monosialylated (20.1%), and asialated (12.0%) variants. Triantennary glycans (those containing five N-acetylglucosamine residues) represent 21.3% of the total, with the predominant forms being fucosylated trisialylated (8.5%), disialylated (6.4%), and monosialylated (3.5%) variants. Tetra-antennary glycans (with six N-acetylglucosamine residues) account for 4.6% of the total glycoforms, consisting primarily of fucosylated trisialylated (1.7%), fucosylated disialylated (1.1%), and other minor sialylated and asialated species.

A key feature of this site is the high degree of sialylation, with approximately 71.5% of all glycans at N108 bearing at least one sialic acid residue. Phosphorylated oligomannose structures constitute a minor but detectable component at this site, comprising approximately 6.1% of the total glycans, which is consistent with the known presence of low-abundance phosphorylated species at sites predominantly occupied by complex glycans.

### 2.4. Glycosylation at N184

The glycan population at N184 is characterized by a heterogeneous mixture of phosphorylated oligomannose, complex, and hybrid structures. A total of 37 distinct glycan species were identified (Figure 3c).

The predominant class of glycoforms, accounting for approximately 36.2% of the total, when excluding the non-glycosylated peptide from the calculation, is constituted by phosphorylated oligomannose glycans (Figure 4c). Mono- and biphosphorylated species account for 20.2% and 16.0% of the total, respectively. These bisphosphorylated species are the most potent ligands for the cation-independent mannose-6-phosphate receptor (CI-MPR) and are critical for efficient lysosomal targeting.

This site also comprises a significant proportion of complex and hybrid structures. Complex-type glycans represent approximately 26.5% of the total glycoforms, with a prevalence of fucosylated, sialylated bi- and tri-antennary structures. The predominant complex species are the monosialylated and disialylated fucosylated biantennary glycans, at 8.2% and 3.0%, respectively, along with their asialated counterpart at 2.3%. Triantennary structures, including both sialylated (N5H5S1 at 8.6%, N5H5S2 at 3.7%) and fucosylated variants, collectively account for approximately 19.4% of the glycoforms. Tetra-antennary structures represent approximately 3.7% of the total.

The phosphorylated and sialylated hybrid glycan N3H7S1P1 was detected at approximately 5.4% relative abundance. Neutral oligomannose structures constitute a minor fraction, accounting for approximately 1.4% of the glycan population. The remaining glycan population consists of various low-abundance species, including high-mannose and complex forms with varying degrees of fucosylation and sialylation.

### 2.5. Glycosylation at N161

A total of 46 distinct glycan species were identified at the N161 site (Figure 3b). The glycan population at N161 is predominantly composed of phosphorylated oligomannose structures. It is noteworthy that a single species, the monophosphorylated oligomannose-7 glycan (N2H7P1), is the most prevalent glycoform, accounting for 42.2% of the total glycans at N161 (Figure 4b). This represents the highest relative abundance of any individual glycan across all three glycosylation sites, thereby underscoring its predominant role at this position. Additional phosphorylated oligomannose species contribute a further 12.4%, while bisphosphorylated oligomannose forms account for 5.5% of the glycoforms.

The high-mannose glycans at N161, both phosphorylated and neutral forms, comprise a diverse array of species that collectively contribute to the site microheterogeneity, although each individual species is present at low abundance. Within this population, phosphorylated glycans predominate substantially over their non-phosphorylated counterparts.

Complex-type glycans account for approximately 30% of the total glycoforms and include a range of sialylated bi- and triantennary structures, primarily in their non-fucosylated forms, along with lower levels of fucosylated variants. The presence of both phosphorylated and non-phosphorylated hybrid structures, along with multiple other glycan classes and numerous low-abundance species, further contributes to site microheterogeneity. This reflects the intrinsic complexity that is characteristic of recombinant glycoproteins produced in mammalian cell systems, despite the clear predominance of phosphorylated oligomannose glycan.

Glycopeptide mapping of Fabrazyme confirmed three N-linked glycosylation sites with highly site-specific glycan distribution. N108 is dominated by highly sialylated complex glycans, N161 by phosphorylated oligomannose, and N184 displays the most heterogeneous profile, containing the highest proportion of bisphosphorylated structures, critical for CI-MPR binding. Over 60 distinct glycan species were identified across all sites, reflecting the intrinsic microheterogeneity of recombinant glycoproteins. To complement this site-specific characterization, intact protein analysis was performed to assess global molecular heterogeneity.

### 2.6. Intact Analysis (Q-ToF)

To the best of our knowledge, no reports have been published to date on the intact MS analysis of full-length Fabrazyme. The deconvoluted mass spectra were annotated using the comprehensive dataset obtained from the two preceding analytical levels: released glycan analysis and site-specific glycopeptide mapping. All possible combinations of N-glycans across the three glycosylation sites were generated using an in-house script, yielding a total of 4862 unique combinations after the removal of duplicates by both glycan name and calculated mass. The resulting combinatorial libraries were imported into the Byos Intact module version 5.10 (Protein Metrics Inc., Boston, MA, USA) for automated matching with the experimental masses, applying a mass tolerance of ±50 ppm.

**Figure 5:**
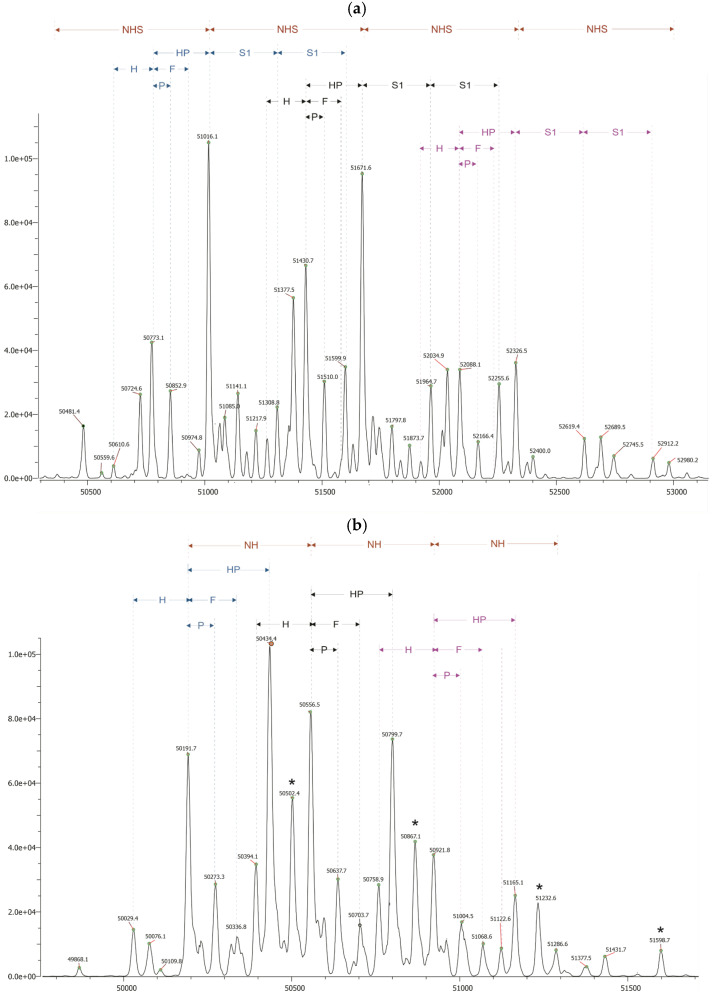
Deconvoluted mass spectra of intact (**a**) and desialylated (**b**) Fabrazyme. The desialylated spectrum (**b**) reveals glycoform series with mass differences corresponding to hexose (H), phosphate (P), phosphorylated hexose (HP), fucose (F), and N-acetylhexosamine plus hexose (NH). In the intact spectrum (**a**), additional increments for sialic acid (S) and sialylated lactosamine (NHS) are observed. * indicate alternative glycoform compositions that form distinct series not following these incremental patterns.

The intact mass profile of desialylated Fabrazyme reveals a series of glycoforms with systematic mass differences that reflect underlying glycan structural variations (Figure 5b). For instance, a recurring increment of approximately 80 Da is observed between pairs such as 50,191.7 Da and 50,273.3 Da, as well as between 50,556.5 Da and 50,637.7 Da, corresponding to the addition of a phosphate group. Increments of approximately 203 Da, indicative of an N-acetylhexosamine residue, are evident between 50,191.7 Da and 50,394.1 Da, and between 50,434.4 Da and 50,637.7 Da. A larger increment of approximately 365 Da, consistent with a lactosamine unit (hexose plus N-acetylhexosamine), distinguishes masses such as 50,191.7 Da from 50,556.5 Da, 50,434.4 Da from 50,799.7 Da, and 50,556.5 Da from 50,921.8 Da. Furthermore, a shift of approximately 242 Da, representing a phosphorylated hexose (hexose plus phosphate), is apparent between 50,191.7 Da and 50,434.4 Da, and between 50,556.5 Da and 50,799.7 Da. These systematic mass differences serve to confirm that the observed heterogeneity arises from combinatorial variations in phosphorylation, antennarity, and lactosamine extension across the three glycosylation sites.

The observed mass differences were further validated by comparing the desialylated and sialylated spectra (Figure 5a). For each major glycoform in the desialylated spectrum, a corresponding peak in the sialylated spectrum was identified, with a mass increase corresponding to a discrete number of sialic acid residues (291 Da per residue). For instance, the desialylated mass of 50,191.7 Da corresponds to the sialylated mass of 50,773.1 Da, indicating a difference of 582 Da and suggesting the presence of two sialic acids. In a similar manner, 50,556.5 Da corresponds to 51,430.7 Da (Δ = 873.7 Da, three sialic acids), and 50,921.8 Da corresponds to 52,088.1 Da (Δ = 1164.6 Da, four sialic acids). These correlations were consistent across the four major series of glycoforms, thereby demonstrating that each lactosamine extension step is accompanied by the addition of one sialic acid residue.

The combinatorial information obtained from glycopeptide mapping served as the basis for assigning site-specific glycan combinations to the experimental intact masses. For the most abundant series, exemplified by the peak at 50,191.7 Da, the combination N4H5F1 (N08) + N2H7P1 (N161) + N2H6P1 (N184) provided the best match with the experimental mass (theoretical 50,189.4 Da, Δ = +2.2 Da). It is important to note that within the ±50 ppm tolerance, alternative assignments are possible. For instance, the same experimental mass could also accommodate the combination N3H4F1 (N108) + N2H7P1 (N161) + N5H5 (N184), yielding the total formula N10H16F1P1 (theoretical 50,191.6 Da). The relative abundances of the constituent glycans from peptide mapping support the former assignment as the predominant contributor. All assignments and relative abundances are provided in Tables S9 and S10.

This ambiguity persists in the sialylated spectra, where overlapping isotopic distributions and the presence of multiple isoforms with similar mass increments further complicate unambiguous discrimination. However, despite the inherent complexity and the impossibility of absolute isoform assignment due to the high microheterogeneity of Fabrazyme, the comprehensive profiling approach presented here provides a valuable framework for structural characterisation. The detailed mass maps and observed regularities offer a robust reference for batch-to-batch consistency assessment and can serve as a sensitive tool for evaluating biosimilarity in comparability studies.

## 3. Discussion

The study of glycosylation of therapeutic proteins such as Fabrazyme (agalsidase beta) presents significant challenges, stemming from both the multiplicity of glycosylation sites and the extensive heterogeneity of glycans attached to each site. For such products, the most commonly applied approaches involve the release of N-glycans followed by fluorescence or mass spectrometric detection and group analysis correlating with therapeutic activity [15]. The method we have developed for the analysis of released and labeled glycans enables rapid and reproducible assessment of the overall glycosylation profile with high sensitivity. Despite technical implementation challenges—including the necessity for a fully bioinert HPLC system, regular passivation of the system and column with phosphoric acid, and an additional sample preparation step—the method was successfully validated and can be recommended for use in quality control laboratories for the release testing of similar products.

In developing the methodology, we built upon previously published data obtained using a Waters C18 AX column for analogous analytes [18]. However, we substantially refined both the chromatographic separation conditions (optimizing mobile phase composition and gradient) and the sample preparation procedure, aimed at preserving labile phosphorylated glycans and effectively removing reaction mixture components. It is worth noting that the C18 AX column exhibits significantly higher selectivity for the retention and separation of these components compared to the amide phase traditionally used for labeled glycan analysis in hydrophilic interaction liquid chromatography (HILIC) mode.

During experiments to optimize conditions for glycopeptide analysis, we demonstrated a substantial advantage of the column based on hybrid technology with BEH (Ethylene Bridged Hybrid) particle chemistry over the CSH (Charged Surface Hybrid) phase when analyzing glycoproteins with complex glycan profiles comprising neutral, phosphorylated, and sialylated glycopeptides. Undoubtedly, for the analysis of fully and multiply sialylated glycoproteins (such as erythropoietin, darbepoetin, tenecteplase, interferons), the use of the CSH phase is justified and can offer significant benefits, particularly when employing elevated concentrations of FA in the mobile phase. Nevertheless, our data indicate the need for further optimization of such conditions to minimize the loss of detection sensitivity.

The utilization of an Orbitrap mass spectrometer (Thermo Fisher Scientific, Waltham, MA, USA) enabled the detection of a significantly higher number of glycoforms at the glycopeptide level compared to released glycan analysis (68 vs. 43 unique glycans, respectively). This is attributed to the higher sensitivity of the Orbitrap instrument operating in reversed-phase liquid chromatography (RP-LC) mode compared with the maXis II 4G (Bruker Corporation, Billerica, MA, USA)operated in mixed-mode reversed-phase/ion-exchange (RP-IEX) chromatography. Although glycopeptide analysis is more labor-intensive and time-consuming during sample preparation compared to released glycan analysis (the latter being substantially simplified by the commercial Waters GlycoWorks kit (Waters Corporation, Milford, MA, USA), it proves to be more cost-effective and, most importantly, provides unique information on site-specific glycan distribution. Such information is critically important, as glycans at different glycosylation sites may exert distinct therapeutic effects.

Intact protein analysis of Fabrazyme presents an even more complex task for unambiguous interpretation. The list we compiled for mass spectrum annotation, based on data obtained from released glycan and glycopeptide analyses, includes over 50 thousand theoretically possible combinations, many of which differ by less than 1 Da. This renders the direct identification of each peak in the intact protein spectrum practically impossible.

Previous attempts to characterize α-galactosidase A at the intact protein level have been reported in the context of glycoengineering studies, where desialylation was likewise employed to reduce spectral complexity [21]. However, those analyses were performed on experimentally engineered agalsidase variants under native MS conditions, and the primary goal was to verify the outcome of glycan remodeling rather than to provide a comprehensive proteoform characterization of the commercial drug substance. In contrast, the present work applies denaturing intact MS to Fabrazyme itself, and introduces a combinatorial library approach—derived from site-specific glycopeptide mapping data—as a systematic framework for proteoform annotation. This integration of glycopeptide-level information into intact spectrum interpretation represents a methodological advance that, to our knowledge, has not been previously reported for this therapeutic.

Nevertheless, the application of a powerful analytical tool such as the maXis II 4G mass spectrometer enables the acquisition of high-resolution spectra suitable for biosimilarity assessment (e.g., when comparing Fabrazyme biosimilars) at any stage of pharmaceutical development. In the obtained results, glycan series characterized by mass shifts corresponding to N-acetylglucosamine, hexose, phosphomannose, and fucose are reliably identified. This, in turn, allows for the inference of gross formula combinations of glycans across the three glycosylation sites. Furthermore, no intact protein species consistent with the ~20% aglycosyl fraction observed at the N184 site at the glycopeptide level were detected. This discrepancy may reflect differential ionisation suppression of sialylated glycoforms at the intact protein level compared with the glycopeptide level. The application of sialidase substantially reduces spectral heterogeneity by removing sialic acid residues, enabling a more unambiguous assessment of the overall glycosylation pattern of the molecule. However, the high resolving power and efficient ion declustering provided by the maXis II 4G mass spectrometer made the key contribution to the quality of the obtained results.

The combination of the methodologies described in this work—released glycan analysis, site-specific glycopeptide mapping, and intact protein analysis—allows for a deep and comprehensive understanding of the glycan profile of complex glycoproteins such as Fabrazyme. The data obtained can be utilized both for comprehensive characterization in biosimilar development and for fundamental research in glycobiology.

## 4. Materials and Methods

### 4.1. Samples

Fabrazyme (ISU Abxis Co., Ltd., Seongnam-si, Republic of Korea; lot no. AI-ISTD001) was purchased from a pharmacy and stored and handled in accordance with the manufacturer’s instructions prior to analysis.

### 4.2. Materials, Chemicals and Reagents

#### 4.2.1. Released Glycan Analysis and MS Annotation

HPLC-grade acetonitrile (ACN) was obtained from Honeywell (Seelze, Germany). Ammonium formate (eluent additive for LC-MS, ≥99.9%), formic acid (FA, LC-MS grade), D-mannitol (BioXtra, ≥98%), sodium dihydrogen phosphate dihydrate (BioUltra, for molecular biology, ≥99.0%), disodium hydrogen phosphate dihydrate (BioUltra, for molecular biology, ≥99.0%), and 4-(2-hydroxyethyl)piperazine-1-ethanesulfonic acid (HEPES, ≥99.5% (titration), for molecular biology, BioUltra) were purchased from Merck (Wicklow, Ireland). Sodium hydroxide (pure, pharma grade) was acquired from PanReac AppliChem (Barcelona, Spain). Proteases and RapiFluor-MS label were obtained from the GlycoWorks Deglycosylation Module and GlycoWorks RapiFluor-MS Labelling Module (Waters Corporation, Milford, MA, USA). Rapigest SF (Waters Corporation, Milford, MA, USA) was used as a surfactant, and TCEP-HCl (Thermo Fisher Scientific, Waltham, MA, USA) was used as a reducing agent.

#### 4.2.2. Glycopeptide Mapping

Rapigest SF (Waters, Ireland) was used as a surfactant. Dithiothreitol (DTT, for molecular biology) was purchased from PanReac AppliChem (Barcelona, Spain), and iodoacetamide (IAA, NMR grade) was obtained from Merck (St. Louis, MO, USA). Ammonium bicarbonate (≥99.0%) and dimethyl sulfoxide (DMSO, LC-MS grade) were obtained from Merck (St. Louis, MO, USA). Trypsin/Lys-C Mix (mass spec grade) was acquired from Promega (Madison, WI, USA).

#### 4.2.3. Intact Analysis

Formic acid (FA, LC-MS grade, ≥99.0%) was acquired from Optima (Acros Organics, Morris Plains, NJ, USA). Trifluoroacetic acid (TFA, LC-MS grade, ≥99.5%) was obtained from Thermo Fisher Scientific (Waltham, MA, USA). α2-3,6,8 Neuraminidase was purchased from New England Biolabs (Ipswich, MA, USA).

### 4.3. Sample Preparation

#### 4.3.1. Released Glycan Analysis and MS Annotation

Deionized water was added to the lyophilizate vial in the volume specified in the drug product instructions. A sample containing 15 µg of protein was diluted with deionized water to a final concentration of 1.5 mg/mL (total volume: 10.0 µL). For denaturation and disulfide bond reduction, 10.0 µL of 3% Rapigest SF and 2.0 µL of 44 mM TCEP were added to the solution. The mixture was thoroughly vortexed and incubated at 90.0 ± 0.1 °C for 3 ± 0.1 min, followed by cooling at room temperature for 3 ± 0.1 min.

GlycoWorks Rapid PNGase F (35 µL) was dissolved in 220.0 µL of deionized water, and 10 µL of this solution was added to the sample. The mixture was incubated at 50.0 ± 0.1 °C for 5 ± 0.1 min. For fluorescent labeling of glycans, 10.0 µL of 82 µg/mL RapiFluor-MS labeling solution was added. The sample was vortexed, centrifuged for 30 s, and incubated at room temperature for 5 ± 0.1 min. Subsequently, 360.0 µL of ACN was added and mixed thoroughly.

A 400.0 µL aliquot of the labeled solution was transferred to a GlycoWorks HILIC µElution Plate. The solution was passed through the sorbent under vacuum. The sorbent was washed three times with a mixture of FA, water, and ACN (1:9:90, *v*/*v*/*v*). Glycans were eluted by adding 30.0 µL of 50 mM sodium citrate solution directly into the plate well.

For additional purification, 90.0 µL of eluate was transferred to a 1.5-mL tube, and 716.0 µL of ACN was added. The resulting solution was transferred to a fresh well of the clean-up plate, and the sorbent washing and glycan elution procedure was repeated as described above. A 90.0-µL aliquot of eluate was transferred to a 0.5-mL LoBind tube (Eppendorf SE, Hamburg, Germany), and 310.0 µL of deionised water was added.

For MS annotation, the lyophilized sample was reconstituted in 30% ACN.

#### 4.3.2. Glycopeptide Mapping

An aliquot corresponding to 30 µg of protein was placed in a microcentrifuge tube and diluted with 100 mM ammonium bicarbonate working solution to a final protein concentration of 4 mg/mL. For disulfide bond reduction, 7.5 µL of 62 mM DTT and 3.0 µL of 0.4% Rapigest solution were added. The mixture was vortexed and incubated at 60 °C for 15 min. For alkylation of free cysteines, 3.0 µL of 180 mM IAA was added, vortexed, and incubated for 30 min at room temperature in the dark.

Subsequently, 1.0 µL of Trypsin/Lys-C Mix (0.25 µg/µL) was added to the aliquot. The sample was vortexed and incubated at 37 °C for 1 h. The enzymatic reaction was terminated by adding 2.0 µL of 1% FA solution.

#### 4.3.3. Intact Analysis

For Intact MS, an aliquot containing 15 µg of protein was transferred to a chromatographic vial. The volume was adjusted to 15 µL with mobile phase A, thoroughly mixed, and subjected to instrumental analysis. For removal of N-acetylneuraminic acid residues, a 10-µg aliquot of protein was diluted with deionised water to 90 µL, and 10 µL of GlycoBuffer 1 and 10 µL of α2-3,6,8 neuraminidase were added. The reaction mixture was mixed and incubated at 37 °C for 16 h.

### 4.4. Instrumental Analysis

#### 4.4.1. Released Glycan Analysis and MS Annotation

Glycan analysis was performed on a Vanquish Flex Binary UHPLC system (Thermo Fisher Scientific, Waltham, MA, USA) equipped with an ACQUITY Premier Glycan BEH C18 AX column (1.7 µm, 2.1 mm × 150 mm) and a matching ACQUITY Premier Glycan BEH C18 AX 95 Å guard column (1.7 µm, 2.1 mm × 5 mm). Validation tests were performed on the Waters Premier binary UPLC system (Waters Corporation, Milford, MA, USA) with FLD-type detection.

The column temperature was maintained at 60 °C. Fluorescence detection was performed at an excitation wavelength of 265 nm and an emission wavelength of 425 nm. Mobile phase A consisted of deionized water, and mobile phase B consisted of 100 mM ammonium formate, pH 4.7, in 50% water/50% ACN. Separation was carried out under gradient elution.

For MS annotation, chromatographic separation was performed on a Thermo Scientific UltiMate 3000 system (Thermo Fisher Scientific, Waltham, MA, USA) coupled to a Bruker maXis II 4G ETD high-resolution Q-ToF mass spectrometer (Bruker Corporation, Billerica, MA, USA) equipped with an electrospray ionization (ESI) source and collision-induced dissociation (CID). Separation was achieved using either a Waters ACQUITY Premier BEH Amide 130Å (Waters Corporation, Milford, MA, USA) or a C18 AX 95Å column (1.7 µm). Mobile phase A was 100 mM ammonium formate, pH 4.7 (±0.01) in water-ACN 50%/50%, and mobile phase B was 100% ACN, using a gradient elution profile.

Detection was performed in positive ESI mode over an *m*/*z* range of 100–2500, with a scan rate of 2 Hz, acquiring both linear and profile spectra. Ion source parameters were set as follows: drying gas flow rate, 9 L/min at 200 °C; capillary voltage, 4500 V; end plate offset, 500 V; nebulizer pressure, 2.2 bar.

#### 4.4.2. Glycopeptide Mapping

Chromatographic separation was performed on a Waters ACQUITY UPLC^®^ system (Waters Corporation, Milford, MA, USA) using an ACQUITY UPLC^®^ Peptide BEH C18 analytical column (130 Å, 1.7 µm, 2.1 mm × 150 mm) equipped with a matching ACQUITY UPLC^®^ Peptide BEH C18 guard cartridge (130 Å, 1.7 µm, 2.1 mm × 5 mm). For comparison, separation was also performed on ACQUITY UPLC^®^ Peptide CSH C18 (130 Å, 1.7 µm, 2.1 mm × 150 mm) with matching guard cartridge. Mobile phase A was 0.1% FA in water, and mobile phase B was 0.1% FA in ACN. The flow rate was set to 0.4 mL/min under gradient elution conditions.

Mass spectrometric detection was carried out on an Orbitrap Exploris 480 (Thermo Fisher Scientific, Waltham, MA, USA) with heated electrospray ionisation (H-ESI) ion source type. Spectra were acquired in positive ion mode using data-dependent acquisition (DDA) over an *m*/*z* range of 200–2000. Ion source parameters were set as follows: capillary voltage, 3800 V; ion transfer tube temperature, 320 °C; vaporizer temperature, 250 °C; sheath gas, 30 Arb; auxiliary gas, 10 Arb; sweep gas, 1 Arb.

#### 4.4.3. Intact MS Analysis

Intact protein analysis was performed on a Thermo Scientific UltiMate 3000 HPLC system (Thermo Fisher Scientific, Waltham, MA, USA) coupled to a Bruker maXis II 4G ETD Q-ToF mass spectrometer (Bruker Corporation, Billerica, MA, USA). Separation was carried out on a Waters ACQUITY BioResolve 2.1 × 50 mm (Waters Corporation, Milford, MA, USA). Gradient elution was performed using mobile phase A (0.1% FA, 0.02% TFA in deionized water) and mobile phase B (0.1% FA in ACN). Detection was performed in positive ESI mode over an *m*/*z* range of 800–4000, with a scan rate of 1 Hz, acquiring both linear and profile spectra. Ion source parameters were set as follows: drying gas flow rate, 10 L/min at 350 °C; capillary voltage, 4500 V; end plate offset, 550 V; nebulizer pressure, 2.5 bar.

### 4.5. Data Analysis

#### 4.5.1. Released Glycan Analysis and MS Annotation

Chromatograms were automatically integrated using Chromeleon 7.4 software. The relative abundance of each glycan group was calculated using the internal normalization method.

MS annotation was performed using Byos Protein Metrics 5.10 software.

#### 4.5.2. Glycopeptide Mapping

Data processing was performed both automatically and manually using the PTM workflow in Byos (Protein Metrics) software.

Peptide identification was based on the following criteria: (1) accurate precursor mass (*m*/*z*), (2) retention time consistency with the expected chromatographic profile, and (3) MS/MS fragmentation patterns. Byonic (Protein Metrics) identifies peptides and glycopeptides by matching experimental MS/MS spectra against theoretical fragments derived from a sequence database. Scoring incorporates precursor and fragment mass accuracy (≤5 ppm for MS1, ≤20 ppm for MS/MS), ion intensity correlation, and, for glycopeptides, the presence of diagnostic oxonium ions and specific glycan neutral losses. Identifications are validated using a target-decoy approach with a false discovery rate of 1% at the peptide-spectrum match level. Confidence is further assessed via posterior error probability.

Theoretical digestion parameters specified trypsin cleavage at the carboxyl side of Lys and Arg residues, except when followed by proline. Glycosylation sites were identified from the list of peptides bearing glycan modifications. The relative abundance of different glycans at each site was determined by the relative amounts of each glycopeptide by the peak area in the extracted ion chromatogram (XIC).

#### 4.5.3. Intact Analysis

Chromatographic peaks corresponding to Fabrazyme were integrated to generate combined mass spectra. These spectra were subsequently deconvoluted to determine the MW of the protein species. The identification of intact masses was carried out using the exact mass.

## 5. Conclusions

The integrated analytical strategy developed in this work—combining released glycan profiling, site-specific glycopeptide mapping, and intact protein mass spectrometry—provides a versatile toolbox for in-depth characterization of complex glycoprotein therapeutics. The complementary information obtained from these orthogonal techniques enables comprehensive assessment of glycosylation heterogeneity, from overall N-glycan distribution to site-specific occupancy and intact proteoform profiling. The established workflow proved particularly valuable for dissecting the intricate glycosylation pattern of Fabrazyme, revealing site-specific features that can influence its biological activity and pharmacokinetic behavior. Beyond this case study, the methodological framework is readily adaptable to other challenging glycoproteins, supporting biosimilar development, batch consistency monitoring, and quality control throughout the product lifecycle. Given the growing interest in biosimilar versions of Fabrazyme and other enzyme replacement therapies, the approach presented here offers a reliable foundation for demonstrating structural and functional comparability. Its versatility also holds potential for application to a wide range of heavily glycosylated biotherapeutics, where detailed glycosylation knowledge contributes to ensuring product quality and clinical performance.

## Data Availability

The data presented in this study are available within the article and its Supplementary Materials. Additional raw data supporting the reported results are available from the corresponding author upon reasonable request.

## Supplementary Materials

The following supporting information can be downloaded at: https://www.mdpi.com/article/10.3390/ijms27083358/s1.
