# Supplementary material for: Integrated Glyco-Analytical Strategy for Comprehensive Characterization of a Complex Therapeutic Glycoprotein: Fabrazyme
Source: Int J Mol Sci. 2026 Apr 8;27(8):3358. doi: 10.3390/ijms27083358 (PMC13115965; doi:10.3390/ijms27083358)
Supplement: Supplementary file 1 [file ijms-27-03358-s001.zip › ijms-4200983-supplementary.pdf]

Integrated glyco-analytical strategy for comprehensive characterization of a complex therapeutic glycoprotein: Fabrazyme

Mikhail Afonin [1]*, Polina Novikova [1], Andrei Vinalev [2] and Natalia Mesonzhnik [1,3]

[1] Resource Centre of Biopharmaceutical Mass-spectrometry, Laboratory Complex, Sirius University of Science and Technology, Olympic Ave. 1, 354340 Sochi, Russia; repin.polina@gmail.com (P.N.); afonin.mb@talantiuspeh.ru (M.A.); natalia.mesonzhnik@gmail.com (N. M.);
[2] Resource Centre of Analytical Method, Laboratory Complex, Sirius University of Science and Technology, Olympic Ave. 1, 354340 Sochi, Russia; vinalev.aa@talantiuspeh.ru (A. V.);
[3] Center for Genetics and Life Sciences, Sirius University of Science and Technology, Olympic Ave. 1, 354340, Sochi, Russia, Russia; natalia.mesonzhnik@gmail.com (N. M.);
* Correspondence: natalia.mesonzhnik@gmail.com;

Supplementary material

>DB00103 sequence

LDNGLARTPTMGWLHWERFMCNLDCQEEPDSCISEKLFMEMAELMVSEGWKDAGYEYLCID
DCWMAPQRDSEGRLQADPQRFPHGIRQLANYVHSKGLKLGIYADVGNKTCAGFPGSFGYYDI
DAQTFADWGVDLLKFDGCYCDSLENLADGYKHMSLALNRTGRSIVYSCEWPLYMWPFQKPN
YTEIRQYCNHWRNFADIDDSWKSIKSILDWTSFNQERIVDVAGPGGWNDPDMLVIGNFGLSW
NQQVTQMALWAIMAAPLFMSNDLRHISPQAKALLQDKDVIAINQDPLGKQGYQLRQGDNFE
VWERPLSGLAWAVAMINRQEIGGPRSYTIAVASLGKGVACNPACFITQLLPVKRKLGFYEWTSR
LRSHINPTGTVLLQLENTMQMSLKDLL

Figure S1. Amino acid sequence of human α-galactosidase A.

The sequence was retrieved from the DrugBank database (accession DB00103, polypeptide sequence file).

Academic Editor: Firstname Lastname

Received: date
Revised: date
Accepted: date
Published: date

Citation: To be added by editorial staff during production.

Copyright: © 2026 by the authors. Submitted for possible open access publication under the terms and conditions of the Creative Commons Attribution (CC BY) license (https://creativecommons.org/licenses/by/4.0/).

Table S1. N-glycan profiling of Fabrazyme labeled with Rapifluor. The table lists identified glycans with their proposed compositions, observed retention times (Apex Time, min), mass-to-charge ratios (Obs. m/z), charge states (z), experimental and calculated monoisotopic masses (Obs. M, Calc. M), and mass accuracy (Δ ppm). Relative abundances are presented as peak area percentages (%).

Apex time	Glycans*	Obs. m/z	z	Obs.M	Calc.M	Δ ppm
21.85	N2H8	1016.8940	2	2031.7734	2031.7665	3.4
	N2H9	1097.9064	2	2193.7982	2193.8193	9.7
22.71	N2H7	935.8648	2	1869.7150	1869.7137	0.7
23.15	N2H6	854.8378	2	1707.6610	1707.6609	0.1
	N2H7	935.8704	2	1869.7263	1869.7137	6.7
24.34	N2H5	773.8122	2	1545.6098	1545.6080	1.1
	N2H4	692.7882	2	1383.5618	1383.5552	4.8
24.90	N2H5	773.8152	2	1545.6159	1545.6080	5.1
	N2H6	854.8428	2	1707.6711	1707.6609	6.0
25.39	N2H4	692.7869	2	1383.5592	1383.5552	2.9
25.78	N3H5	875.3518	2	1748.6891	1748.6874	1.0
26.20	N2H3	611.7662	2	1221.5177	1221.5024	12.6
26.56	N3H4	794.3254	2	1586.6363	1586.6346	1.1
27.55	N4H4	895.8587	2	1789.7028	1789.7140	6.2
	N4H5	976.8801	2	1951.7457	1951.7668	10.8
29.50	N5H5F1	767.9746	3	2300.9019	2300.9041	0.9
	N5H6F1	821.9887	3	2462.9444	2462.9569	5.1
29.92	N2H9P1	1137.8866	2	2273.7587	2273.7857	11.8
	N6H7F1	943.7068	3	2828.0984	2828.0891	3.3
30.84	N2H8P1	1056.8617	2	2111.7089	2111.7328	11.3
31.68	N2H7P1	975.8522	2	1949.6899	1949.6800	5.1
32.14	N2H6P1	894.8252	2	1787.6359	1787.6272	4.9
	N2H7P1	975.8509	2	1949.6873	1949.6800	3.8
32.57	N2H6P1	894.8232	2	1787.6319	1787.6272	2.6
33.07	N2H5P1	813.7967	2	1625.5788	1625.5744	2.7
	N2H6P1	894.8256	2	1787.6366	1787.6272	5.2
33.44	N2H5P1	813.7962	2	1625.5779	1625.5744	2.2
	N2H6P1	894.8252	2	1787.6359	1787.6272	4.8
33.83	N2H5P1	813.7962	2	1625.5777	1625.5744	2.1

Apex time	Glycans*	Obs. m/z	z	Obs.M	Calc.M	Δ ppm
	N2H6P1	894.8252	2	1787.6359	1787.6272	4.8
34.54	N2H6P1	894.8120	2	1787.6094	1787.6272	9.9
34.99	N3H6S1	734.9552	3	2201.8439	2201.8357	3.7
36.06	N3H5S1	680.9375	3	2039.7906	2039.7828	3.8
36.36	N3H5S1	680.9338	3	2039.7796	2039.7828	1.6
36.79	N4H5S1	748.6270	3	2242.8591	2242.8622	1.4
37.34	N4H4S1	1041.4028	2	2080.7910	2080.8094	8.8
	N6H7F1S1	1040.7240	3	3119.1503	3119.1845	11.0
37.84	N5H6F1S1	919.0130	3	2754.0172	2754.0523	12.7
	N6H7F1S1	780.7941	4	3119.1474	3119.1845	11.9
38.29	N5H6F1S1	919.0156	3	2754.0249	2754.0523	10.0
39.35	N4H5F1S1	797.3118	3	2388.9136	2388.9201	2.7
	N2H8P2	1096.8447	2	2191.6748	2191.6992	11.1
40.17	N4H5F1S1	797.3154	3	2388.9245	2388.9201	1.8
40.41	N2H7P2	677.5602	3	2029.6589	2029.6463	6.2
41.51	N3H7S1P1	815.6274	3	2443.8603	2443.8548	2.2
42.49	N2H6P2	934.7941	2	1867.5737	1867.5935	10.6
43.74	N5H6S2	967.3602	3	2899.0587	2899.0898	10.7
	N7H8F1S2	944.8648	4	3775.4301	3775.4121	4.8
44.43	N4H5S2	1267.9795	2	2533.9444	2533.9576	5.2
44.90	N5H6F1S2	1016.0466	3	3045.1178	3045.1477	9.8
46.77	N5H6F1S2	1016.0614	3	3045.1622	3045.1477	4.8
46.97	N4H5F1S2	894.3491	3	2680.0255	2680.0155	3.7
47.70	N5H5F1S2	962.0420	3	2883.1043	2883.0949	3.3
48.03	N4H5F1S2	894.3349	3	2679.9828	2680.0155	12.2
48.77	N5H5F1S2	962.0285	3	2883.0638	2883.0949	10.8
	N5H6S3	1064.3884	3	3190.1435	3190.1852	13.1
49.11	N5H6S2Sg1	1069.7360	3	3206.1862	3206.1801	1.9
	N7H8F1S3	1017.6243	4	4066.4681	4066.5075	9.7

Apex time	Glycans*	Obs. m/z	z	Obs.M	Calc.M	Δ ppm
49.73	N6H7F1S3	926.3553	4	3701.3920	3701.3753	4.5
50.31	N6H7F1S3	926.3417	4	3701.3378	3701.3753	10.1
51.08	N5H6F1S3	1113.0801	3	3336.2185	3336.2431	7.4
51.77	N5H6F1S3	1113.0812	3	3336.2217	3336.2431	6.4
52.47	N5H6F1S3	835.0710	4	3336.2550	3336.2431	3.5
53.54	N5H6F1S3	1113.0800	3	3336.2181	3336.2431	7.5
54.44	N7H8F1S4	1090.3993	4	4357.5680	4357.6029	8.0
55.91	N6H7F1S4	999.1299	4	3992.4904	3992.4708	4.9
56.37	N6H7F1S4	999.1099	4	3992.4106	3992.4708	15.1
57.37	N6H7F1S4	999.1151	4	3992.4314	3992.4708	9.9

*The following abbreviations for monosaccharide residues and modifications in glycan structures: N – N-acetylhexosamine (HexNAc); H – hexose (mannose, galactose); F – fucose; S – N-acetylneuraminic acid (NeuAc); Sg – N-glycolylneuraminic acid (NeuGc); P – phosphate. The numeral following each symbol denotes the quantity of the residue in question within the glycan structure.

Definition of glycan groups used in validation studies

For validation purposes, glycans were grouped into eight categories* based on their structural features, as detailed below:

1. HM (neutral oligomannose glycans): contain no phosphate and no sialic acid. Representative compositions: N2H3, N2H4, N2H5, N2H6, N2H7, N2H8, N2H9.
2. N (neutral complex/hybrid glycans): contain no phosphate and no sialic acid. Representative compositions: N3H4, N3H5, N4H4, N4H5, N5H5F1, N5H6F1, N6H7F1.
3. 1-P (monophosphorylated glycans): contain one phosphate group, with or without sialic acid. Representative compositions: N2H5P1, N2H6P1, N2H7P1, N2H8P1, N2H9P1, N3H7S1P1.
4. 2-P (bisphosphorylated glycans): contain two phosphate groups. Representative compositions: N2H6P2, N2H7P2, N2H8P2.
5. 1-SA (monosialylated glycans): contain one sialic acid and no phosphate. Representative compositions: N3H5S1, N3H6S1, N4H4S1, N4H5S1, N5H6F1S1, N6H7F1S1.
6. 2-SA (disialylated glycans): contain two sialic acids and no phosphate. Representative compositions: N4H5S2, N5H5F1S2, N5H6F1S2, N5H6S2, N7H8F1S2.
7. 3-SA (trisialylated glycans): contain three sialic acids and no phosphate. Representative compositions: N5H6F1S3, N6H7F1S3, N7H8F1S3.
8. 4-SA (tetrasialylated glycans): contain four sialic acids and no phosphate. Representative compositions: N6H7F1S4, N7H8F1S4.

***Note:** Glycans containing both phosphate and sialic acid (e.g., N3H7S1P1) are included in the phosphorylated groups (1-P or 2-P).

Table S2. Intra-laboratory repeatability of the HPLC-FLD method for N-glycan profiling of Fabrazyme (Analyst 1). Data represent relative peak area percentages for each glycan group from six independent injections. Mean, standard deviation (SD), relative standard deviation (RSD, %), and p-values for Dixon's outlier test and Anderson-Darling normality test are shown.

**Run**	**HM (%)**	**N (%)**	**1-P (%)**	**1-SA (%)**	**2-P (%)**	**2-SA (%)**	**3-SA (%)**	**4-SA (%)**
1	2.86	0.95	28.40	16.49	6.31	28.65	12.26	4.10
2	2.85	0.95	28.40	16.41	6.32	28.69	12.29	4.08
3	2.85	0.94	28.41	16.43	6.30	28.70	12.26	4.12
4	2.86	0.95	28.38	16.40	6.33	28.72	12.30	4.08
5	2.89	0.95	28.41	16.39	6.32	28.69	12.25	4.10
6	2.87	0.95	28.39	16.34	6.31	28.76	12.27	4.11
Mean	2.86	0.95	28.40	16.41	6.32	28.65	12.26	4.10
SD	0.015	0.005	0.012	0.049	0.011	0.037	0.019	0.016
RSD (%)	0.50	0.50	0.04	0.30	0.20	0.10	0.20	0.40
Dixon's r11 p-value	0.404	0.099	0.853	0.215	0.404	0.262	1.000	1.000
Anderson-Darling p-value	0.220	0.138	0.428	0.634	0.607	0.551	0.38	0.414

Table S3. Intra-laboratory repeatability of the HPLC-FLD method for N-glycan profiling of Fabrazyme (Analyst 2). Data represent relative peak area percentages for each glycan group from six independent injections. Mean, SD, RSD, and p-values for Dixon's and Anderson-Darling tests are provided.

**Run**	**HM (%)**	**N (%)**	**1-P (%)**	**1-SA (%)**	**2-P (%)**	**2-SA (%)**	**3-SA (%)**	**4-SA (%)**
7	3.40	1.02	28.21	16.35	6.26	28.73	12.09	3.93
8	3.50	1.01	28.14	16.31	6.27	28.71	12.10	3.96
9	3.48	0.98	28.11	16.36	6.19	28.80	12.12	3.96
10	3.49	1.00	28.09	16.29	6.21	28.85	12.12	3.93
11	3.45	1.02	28.10	16.32	6.21	28.81	12.13	3.95
12	3.43	0.99	27.97	16.26	6.28	28.87	12.20	4.00
Mean	3.46	1.00	28.10	16.32	6.24	28.80	12.13	3.96
SD	0.039	0.016	0.078	0.037	0.038	0.064	0.039	0.026
RSD (%)	1.12	1.63	0.28	0.23	0.61	0.22	0.32	0.65
Dixon's r11 p-value	0.853	1.000	0.086	0.853	1.000	1.000	0.092	0.262
Anderson-Darling p-value	0.637	0.573	0.361	0.881	0.217	0.555	0.095	0.267

Table S4. Intra-laboratory repeatability (n = 12) and intermediate precision statistics. Mean, SD, RSD, and results of F-test (Standard Deviation Test) and t-test between Analyst 1 and Analyst 2 are shown. The ±3σ acceptance limits are also provided.

**Parameter**	**HM (%)**	**N (%)**	**1-P (%)**	**1-SA (%)**	**2-P (%)**	**2-SA (%)**	**3-SA (%)**	**4-SA (%)**
Mean (n=12)	3.17	0.97	28.25	16.36	6.28	28.75	12.2	4.03
SD	0.312	0.033	0.163	0.065	0.049	0.07	0.081	0.078
RSD (%)	9.90	3.30	0.60	0.40	0.80	0.20	0.70	1.90
Standard Deviation Test (p-value)	0.089	0.021	0.007	0.558	0.002	0.223	0.347	0.338
t-test (p-value)	0.001	0.001	0.001	0.017	0.004	0.017	0.001	0.001
Upper limit (+3σ)	4.11	1.07	28.74	16.55	6.43	28.96	12.44	4.26
Lower limit (-3σ)	2.23	0.87	27.76	16.17	6.13	28.54	11.96	3.8

Table S5. Intra-laboratory transfer study. Results from six independent injections performed in a second HPLC-FLD. Mean, SD, RSD, and p-values for Dixon's and Anderson-Darling tests are presented.

**Run**	**HM (%)**	**N (%)**	**1-P (%)**	**1-SA (%)**	**2-P (%)**	**2-SA (%)**	**3-SA (%)**	**4-SA (%)**
1	2.84	0.96	28.24	15.85	7.25	28.46	12.53	3.87
2	2.89	0.92	27.90	15.92	7.21	28.61	12.60	3.86
3	2.87	0.97	28.21	15.84	7.26	28.23	12.59	3.79
4	2.94	0.99	28.15	15.84	7.27	28.11	12.57	3.79
5	2.92	1.03	28.28	15.90	7.07	28.05	12.44	3.71
6	2.89	1.06	27.90	15.88	7.10	27.90	12.37	4.00
Mean	2.89	0.99	28.11	15.87	7.19	28.23	12.52	3.84
SD	0.035	0.050	0.171	0.034	0.087	0.266	0.092	0.099
RSD (%)	1.23	5.10	0.61	0.21	1.21	0.94	0.74	2.57
Dixon's r11 p-value	0.727	0.761	1.000	1.000	1.000	1.000	0.900	0.186
Anderson-Darling p-value	0.838	0.885	0.096	0.335	0.115	0.780	0.258	0.540

Table S6. Glycopeptide* identifications and relative abundances for the Asn-108 glycosylation site of Fabrazyme.

Glycan	Apex Time (min)	Relative Abundance (%)
N4H5F1S2	13.67	27.842
N4H5F1S1	13.12	11.777
N5H6F1S3	13.84	8.527
N4H5F1S1	13.13	8.273
N4H5F1	12.57	6.714
N5H6F1S2	13.53	6.400
N3H4F1S1	13.67	4.769
N5H6F1S1	13.00	3.508
N2H6P1	13.33	3.292
N4H3F1	12.74	2.598
N2H5P1	13.43	1.790
N6H7F1S3	13.70	1.696
N4H4F1	12.68	1.584
N6H7F1S2	13.29	1.105
N4H4F1	12.68	1.074
N2H4P1	13.53	1.035
N2H5	12.68	0.986
N2H3F1	12.90	0.959
N2H2F1	13.01	0.913
N5H6F1	12.44	0.817
N5H5F1S1	13.07	0.785
N6H7F1S4	13.97	0.695
N5H5F1	12.51	0.693
N5H5S2	13.89	0.431
N6H7F1S1	12.82	0.412
N6H6S3	12.94	0.252
N6H3F1	12.59	0.144
N2H4	12.79	0.141
N5H5S2	13.91	0.130

N6H7F1	12.26	0.123

*The deamidated aglycosyl peptide (N108_Dea) was detected at 0.382% and is not included in the glycan list.

Table S7. Glycopeptide identifications and relative abundances for the Asn-161 glycosylation site of Fabrazyme.

Glycan	Apex Time (min)	Relative Abundance (%)
N2H7P1	11.70	42.194
N4H5S1	11.72	8.731
N4H5S2	12.31	8.228
N2H6P1	11.80	6.514
N2H8P1	11.66	3.344
N4H5	10.83	2.944
N3H7S1P1	12.37	2.283
N4H5F1S2	12.37	2.237
N2H5	10.86	1.925
N4H5F1S1	11.78	1.888
N2H6	10.76	1.848
N2H7P2	12.86	1.411
N2H8P2	12.76	1.389
N2H5P1	11.80	1.015
N2H9P1	11.61	0.949
N2H9P2	12.72	0.895
N5H5S2	8.45	0.769
N5H5	7.36	0.676
N4H5F1	10.90	0.658
N3H6S1	11.43	0.585
N4H5S2	12.32	0.582
N4H4	10.94	0.574
N3H5S1	11.54	0.555
N2H7	10.71	0.549
N5H6S2	12.13	0.488

N2H4P1	12.04	0.414
N3H6	10.78	0.380
N5H5S1	7.71	0.349
N3H5	10.88	0.333
N5H6S1	11.30	0.332
N3H7S1P1	12.38	0.306
N2H8P2	12.75	0.300
N5H5S2	8.47	0.253
N4H4F1	11.02	0.159
N4H3	10.99	0.129
N4H4S1	11.59	0.129
N5H6F1S2	12.18	0.120
N2H8	10.65	0.114
N5H6F1S3	12.81	0.104
N4H4	10.95	0.103
N5H6F1S1	11.35	0.092
N3H6S1	11.44	0.069
N2H9	10.61	0.061
N5H6	10.65	0.056
N3H4F1	11.53	0.051
N2H8	10.64	0.028

*The aglycosyl peptide (N161_agly) and its deamidated form (N161_dea) were detected at 2.538% and 0.350%, respectively, and are not included in the glycan list.

Table S8. Glycopeptide identifications and relative abundances for the Asn-184 glycosylation site of Fabrazyme.

Glycan	Apex Time (min)	Relative Abundance (%)
N2H6P1	25.59	17.178
N2H7P2	26.57	11.378
N4H5F1S1	25.12	8.244
N5H5S1	21.68	7.781
N5H5S2	22.45	3.654

N2H6P2	26.64	3.388
N4H5F1S2	25.96	3.016
N3H7S1P1	26.24	2.729
N3H7S1P1	26.23	2.641
N4H5F1	24.32	2.312
N5H5	20.99	1.953
N5H6F1S2	25.75	1.522
N6H6S2	22.28	1.488
N2H5P1	25.67	1.247
N2H4P1	25.73	0.990
N2H5	24.53	0.980
N5H6F1S1	25.01	0.868
N5H5S1	21.69	0.867
N5H4	21.05	0.824
N6H6S1	21.61	0.764
N6H6S3	22.99	0.691
N4H4F1	24.40	0.539
N5H3	21.08	0.489
N5H6F1S3	26.48	0.472
N3H3F1	24.51	0.447
N6H5F1	22.02	0.425
N4H4F1	24.39	0.406
N2H7P1	25.53	0.388
N2H4P1	25.73	0.341
N4H3	21.13	0.328
N3H4F1	25.12	0.319
N5H5F1S2	25.73	0.271
N5H5	20.98	0.236
N2H7	24.42	0.192
N3H4F1S1	25.96	0.158
N6H6	20.92	0.151

N3H3	21.18	0.067

*The aglycosyl peptide (N184_agly) was detected at 19.461% and is not included in the glycan list. A deamidated peptide corresponding to Q181 was detected at 0.341% and represents a peptide modification rather than a glycosylation variant.

a)

N108 N4H5F1S2

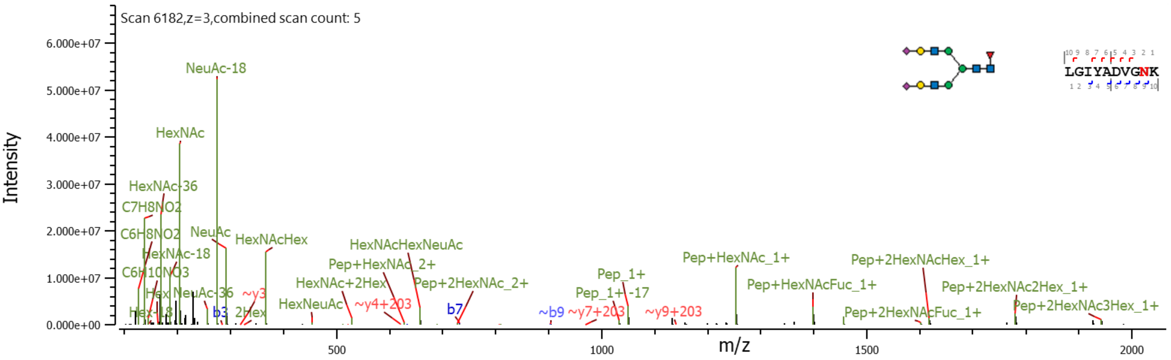

b)

N108 N4H5F1S1

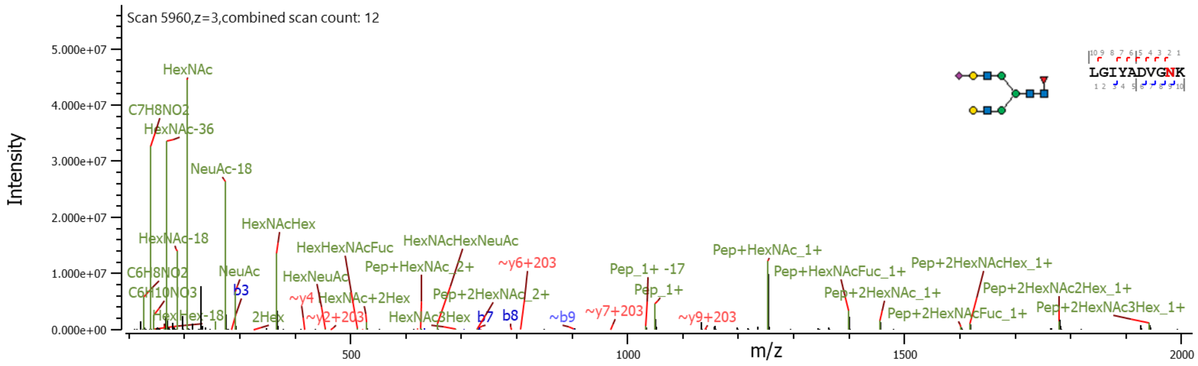

c)

N108 N4H5F1

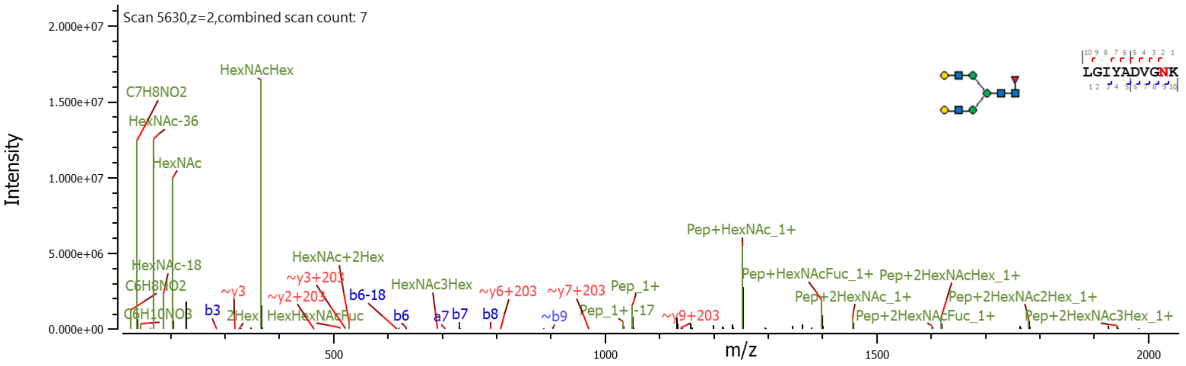

d)

N161 N2H8P1

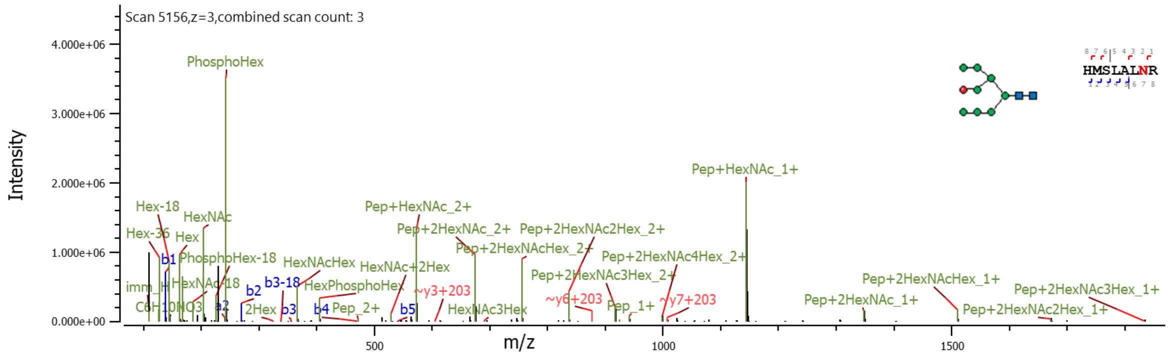

Figure S2. Annotated MS/MS spectra of representative glycopeptides from each N-glycosylation site of Fabrazyme**(a-d).**

e)

N161 N2H7P1

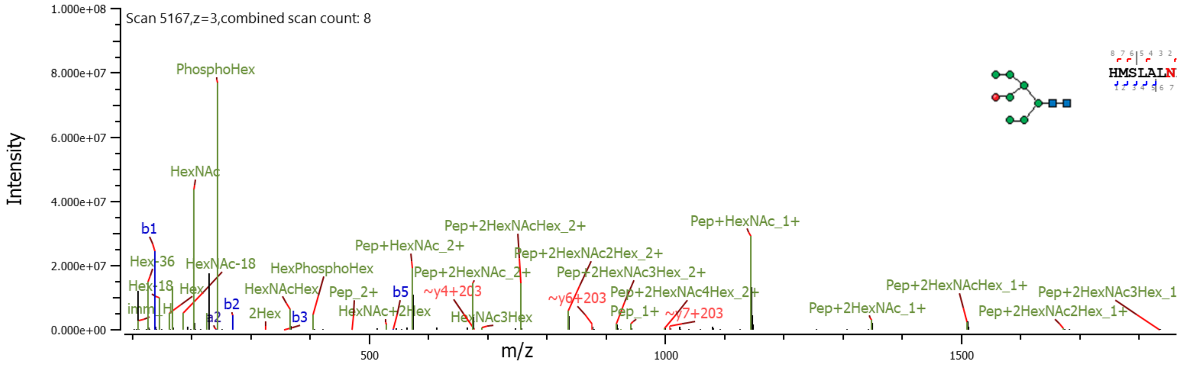

f)

N161 N3H7S1P1

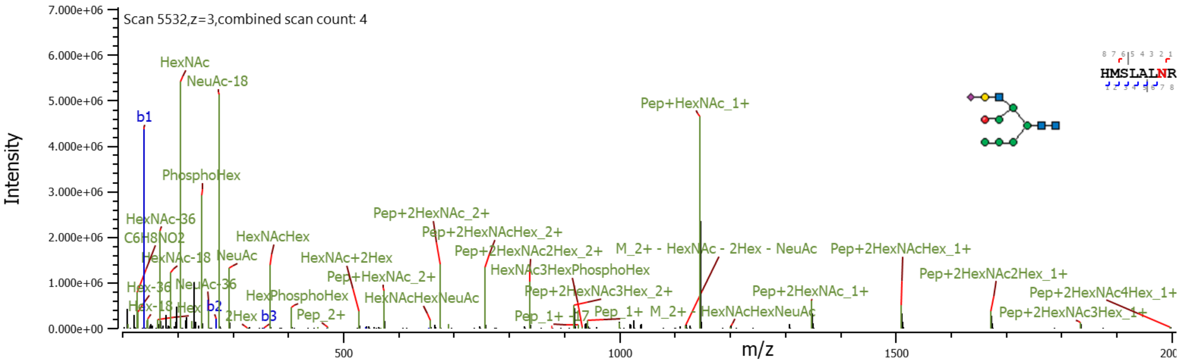

g)

N184 N2H6P1

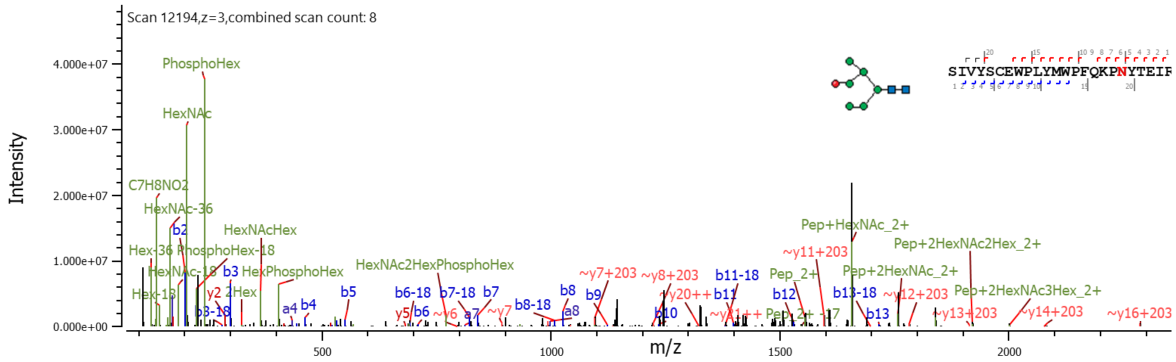

h)

N184 N2H7P2

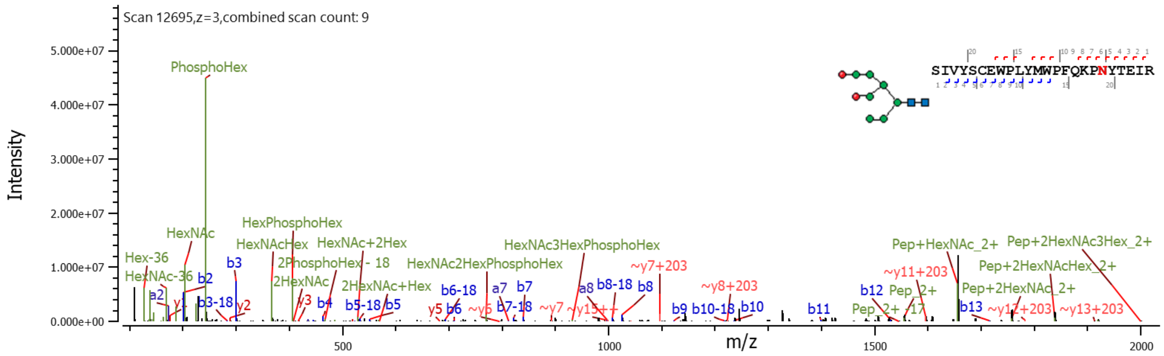

Figure S3. Annotated MS/MS spectra of representative glycopeptides from each N-glycosylation site of Fabrazyme**(e-h)**.

i)

N184 N4H5F1S1

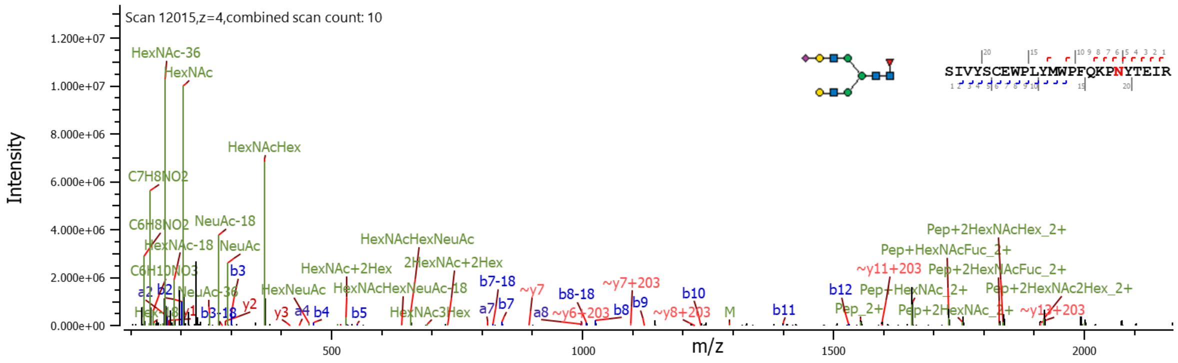

j)

N184 N5H5F1

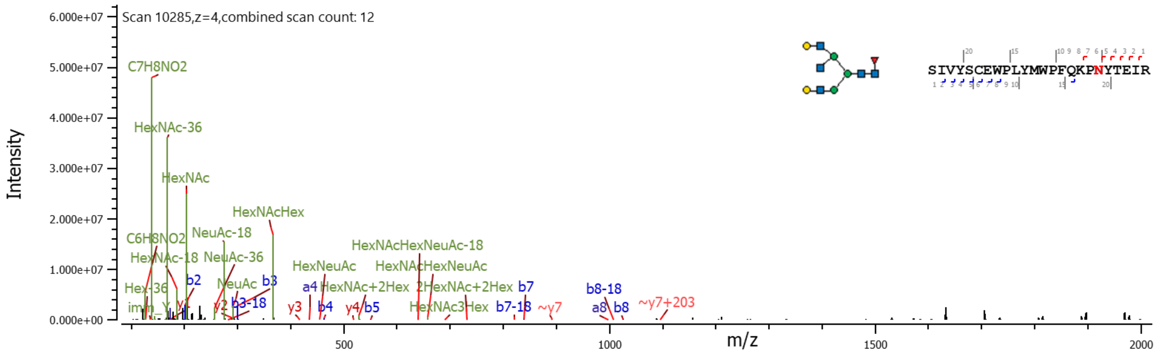

Figure S4. Annotated MS/MS spectra of representative glycopeptides from each N-glycosylation site of Fabrazyme**(i-j).**

*Note: For each of the three glycosylation sites, the most abundant glycopeptide species representing distinct structural classes are shown. Fragmentation spectra were acquired by LC-MS/MS with CID. Diagnostic oxonium ions (e.g., m/z 204.1, 366.1), peptide backbone fragments (b/y ions), and glycan-neutral losses are indicated, confirming both peptide identity and glycan composition

Table S9. Detected intact masses of Fabrazyme and putative mass assignments within 50 ppm mass accuracy.

Average Mass (Da)	Mass annotation variants	%
50481.4	N8H18F1S1P2 // N12H13F1S1P2 // N10H16F1S1P1 // N8H19F1S1 // N10H12F2S3 // N15H12F1	1.7
50559.6	N13H15F1 // N8H18F1S1P3 // N11H13F4S1	0.2
50610.6	N8H17F1S2P2 // N10H19P2 // N10H15F1S2P1 // N8H18F1S2 // N15H11F1S1	0.4
50724.6	N10H17F1S1P2 // N8H20F1S1P1 // N10H13F2S3P1 // N8H19F1S1P3	2.7
50773.1	N10H20P2 // N10H16F1S2P1 // N8H18F1S2P2 // N8H19F1S2 // N10H12F2S4 // N15H12F3	4.3
50852.9	N8H18F1S2P3 // N10H20P3 // N10H16F1S2P2 // N8H19F1S2P1 // N13H15F3 // N11H13F6S1 // N13H15F1S1	2.8
50974.8	N16H12F3 // N9H18F1S2P2 // N16H12F1S1 // N11H16F1S2P1	0.9
51016.1	N10H21P3 // N10H17F1S2P2 // N8H20F1S2P1 // N8H19F1S2P3 // N12H15F1S2P1 // N10H18F1S2	10.6
51064.2	N10H20S1P2 // N10H16F1S3P1	1.7
51085.0	N12H15F2S2 // N10H22F1 // N12H19F1P1 // N12H15F4S1 // N14H16F1P2 // N10H13F5S3 // N12H15S3 // N10H17F4S1P1 // N14H17F1 // N10H13F3S4	1.9
51141.1	N13H15F1S2 // N11H13F6S2 // N11H13F4S3 // N13H15F3S1 // N11H13F2S4 // N9H20F1S2 // N11H17F1S2P1 // N13H14F1S2P2	2.7
51178.8	N10H14F2S4P1 // N8H21F1S2P1 // N10H18F1S2P2 // N12H16F1S2P1 // N10H19F1S2 // N12H12F2S4 // N8H20F1S2P3	0.8
51217.9	N9H19F1S2P3 // N14H16F3 // N11H17F1S2P2 // N9H20F1S2P1 // N12H14F6S1 // N14H16F1S1	1.5
51266.0	N9H18F1S3P2 // N16H12F1S2 // N9H18F5S1P2	1.3
51308.8	N12H15F1S3P1 // N10H18F1S3 // N10H17F1S3P2 // N10H21S1P3 // N12H19F2P2 // N12H15F3S2P1	2.3
51358.4	N12H18S2P1 // N10H16F5S2P1 // N12H14F1S4 // N10H16F3S3P1 // N12H18F2S1P1 // N12H14F3S3 // N10H16F1S4P1 // N10H20S2P2 // N14H16S2 // N12H14F5S2 // N17H14F2	1.7
51377.5	N14H17F1S1 // N12H15F6S1 // N12H15F4S2 // N14H17F3 // N12H15F2S3 // N10H22F1S1 // N12H19F1S1P1 // N14H16F1S1P2 // N9H20F1S2P3	5.7
51430.7	N11H17F1S3P1 // N9H20F1S3 // N11H13F2S5 // N11H17F3S2P1 // N9H19F1S3P2 // N11H17F5S1P1 // N13H15F1S3 // N11H13F6S3	6.8
51510.0	N11H17F1S3P2 // N9H20F1S3P1 // N9H19F1S3P3 // N11H17F3S2P2 // N14H16F3S1	3.1
51599.9	N10H17F3S3P2 // N12H19S2P2 // N10H17F5S2P2 // N12H15F1S4P1 // N10H18F1S4 // N10H17F1S4P2 // N10H21S2P3 // N15H18F2 // N12H19F2S1P2 // N12H15F3S3P1 // N10H18F3S3	3.5
51632.3	N10H19F1S3P2 // N12H17F1S3P1 // N10H20F1S3 // N12H13F2S5 // N12H21F2P2	1.1

51671.6	N9H20F1S3P3 // N11H18F1S3P2 // N9H21F1S3P1 // N14H17F3S1 // N11H18F3S2P2 // N12H15F6S2 // N14H17F1S2	9.7
51716.4	N12H18F1S3 // N14H15F1S3P1 // N14H19F2P2 // N14H15F3S2P1 // N12H18F3S2 // N12H17F3S2P2 // N12H18F5S1 // N16H13F1S3 // N10H20F1S3P1 // N12H17F1S3P2	2.0
51743.3	N13H16F6S1 // N15H18F1S1 // N10H21F1S2P3 // N10H17F2S4P2 // N13H16F2S3 // N13H20F1S1P1	1.6
51797.8	N12H18F5S1P1 // N14H16F1S3 // N12H14F6S3 // N12H14F4S4 // N12H18F3S2P1 // N14H20F2P1 // N14H16F3S2 // N12H14F2S5 // N10H21F1S3 // N12H18F1S3P1 // N14H16F5S1	1.6
51834.8	N11H19F1S3P2 // N9H22F1S3P1 // N11H15F2S5P1 // N11H19F3S2P2 // N11H15F4S4P1 // N9H21F1S3P3 // N11H19F5S1P2 // N13H17F1S3P1 // N11H20F1S3	0.6
51873.7	N10H20F1S3P3 // N15H17F3S1 // N13H15F6S2 // N15H17F1S2 // N12H18F1S3P2 // N13H15F4S3 // N10H21F1S3P1	1.0
51921.4	N13H18F5S1 // N10H19F1S4P2 // N13H18F1S3 // N11H20F5S1P1 // N13H17F5S1P2	0.5
51964.7	N11H18F3S3P2 // N11H18F1S4P2 // N13H20S2P2 // N11H18F5S2P2 // N13H16F1S4P1 // N11H19F1S4 // N14H17F5S1 // N13H20F2S1P2 // N13H16F3S3P1	2.9
52013.7	N11H17F1S5P1 // N11H17F3S4P1 // N13H19S3P1 // N11H17F5S3P1 // N13H15F1S5 // N13H19F2S2P1	1.5
52034.9	N13H16F6S2 // N15H18F1S2 // N15H18F3S1 // N10H21F1S3P3 // N13H16F4S3 // N13H16F2S4	3.4
52088.1	N12H18F3S3P1 // N12H14F4S5 // N10H21F1S4 // N12H18F1S4P1 // N12H18F5S2P1 // N14H16F1S4 // N12H14F6S4 // N14H20F2S1P1 // N14H16F3S3	3.4
52166.4	N15H17F5S1 // N12H18F1S4P2 // N10H21F1S4P1 // N10H20F1S4P3 // N15H17F3S2 // N12H18F3S3P2	1.2
52255.6	N11H18F1S5P2 // N11H18F3S4P2 // N16H19F2S1 // N13H20S3P2 // N11H18F5S3P2 // N13H16F1S5P1 // N11H19F1S5 // N14H17F5S2	3.0
52294.2	N15H16F5S2 // N15H16F3S3 // N17H18F2S1 // N13H14F6S4 // N15H16F1S4 // N13H18F5S2P1 // N12H17F1S5P2	0.5
52326.5	N15H18F3S2 // N10H21F1S4P3 // N13H16F6S3 // N15H18F1S3 // N13H20F5S1P1 // N15H18F5S1 // N13H16F4S4	3.7
52375.8	N17H14F3S3 // N17H14F1S4 // N13H19F5S2 // N13H19F3S3	0.5
52400.0	N14H17F6S2 // N14H17F4S3 // N11H22F1S3P3 // N11H18F2S5P2 // N14H17F2S4 // N14H21F1S2P1	0.7
52451.8	N13H19F1S4P1 // N13H15F2S6 // N11H21F1S4P2 // N13H19F3S3P1 // N13H15F4S5	0.1
52619.4	N15H18F5S2 // N12H19F1S5P2 // N15H18F3S3 // N12H19F3S4P2 // N13H16F6S4 // N15H18F1S4 // N13H20F5S2P1	1.3
52689.5	N14H17F4S4 // N14H17F2S5 // N14H21F1S3P1 // N14H17F6S3 // N14H17S6 // N12H19F4S4P1 // N16H19F3S2	1.3
52745.5	N13H19F5S3P1 // N13H15F4S6 // N15H17F1S5 // N13H19F3S4P1 // N15H17F3S4 // N13H19F1S5P1 // N15H17F5S3	0.7
52912.2	N12H19F1S6P2 // N12H19F3S5P2 // N14H21S4P2 // N12H19F5S4P2 // N14H17F1S6P1 // N15H18F5S3	0.6
52980.2	N14H17F2S6 // N14H21F1S4P1 // N14H21F3S3P1 // N14H17F4S5 // N14H17F6S4 // N12H19F2S6P1	0.5

Table S10. Detected Intact Masses of Fabrazyme and Putative Mass Assignments within 50 ppm Mass Accuracy Following Treatment with Neuraminidase A.

**Average Mass (Da)**	**Mass annotation variants**	**%**
48732.9	N8H10F1	0.4
48934.8	N9H10F1 // N7H12F1P1	0.1
49244.0	N9H11F2 // N7H13F2P1	0.1
49868.1	N8H17F1 // N10H14F1P1 // N12H12F1	0.3
50029.4	N10H15F1P1 // N12H12F1P2 // N8H18F1 // N8H17F1P2 // N12H13F1 // N10H14F1P3	1.7
50109.8	N8H18F1P1 // N10H15F1P2 // N12H13F1P1 // N10H16F1	0.2
50191.7	N8H19F1 // N10H16F1P1 // N12H13F1P2 // N12H14F1 // N10H12F6 // N8H18F1P2	8.0
50229.6	N9H17F1P2 // N7H20F1P1 // N7H19F1P3	1.3
50273.3	N12H14F1P1 // N10H17F1 // N8H19F1P1 // N10H16F1P2	3.3
50320.1	N10H15F3P1	1.2
50336.8	N12H13F2P2 // N10H16F2P1	1.4
50352.0	N8H19F1P2 // N10H16F1P3 // N12H14F1P2 // N10H17F1P1 // N8H20F1	0.8
50394.1	N13H13F1P2 // N11H16F1P1 // N9H19F1 // N9H18F1P2	4.0
50434.4	N8H20F1P1 // N10H17F1P2 // N12H15F1P1 // N10H18F1	11.9
50477.6	N11H17F1 // N13H14F1P1 // N15H12F1	1.3
50502.4	N12H15F2 // N14H12F2P1	6.4
50556.5	N13H14F1P2 // N11H17F1P1 // N9H20F1 // N9H19F1P2 // N11H16F1P3	9.6
50577.0	N11H14F5 // N13H16	2.0
50596.0	N8H21F1P1 // N10H18F1P2 // N12H16F1P1 // N10H19F1 // N8H20F1P3	2.1
50637.7	N9H20F1P1 // N11H17F1P2 // N13H15F1P1 // N11H18F1	3.5
50684.9	N11H16F3P1	0.5
50703.7	N11H17F2P1 // N13H15F2	1.9
50713.8	N9H19F1P4 // N10H17F4	1.1
50758.9	N10H19F1P2 // N12H17F1P1 // N10H20F1	3.3

50799.7	N9H21F1P1 // N11H18F1P2 // N13H16F1P1 // N11H19F1	8.6
50839.5	N12H17F1P2 // N10H20F1P1 // N10H19F1P3	0.7
50867.1	N13H16F2 // N11H18F2P1 // N13H15F2P2	4.9
50904.9	N10H19F2P2 // N12H17F2P1 // N10H20F2	0.8
50921.8	N12H18F1P1 // N10H21F1 // N10H20F1P2	4.4
50959.9	N9H21F1P3 // N11H19F1P2 // N9H22F1P1	1.3
51004.5	N12H19F1 // N14H16F1P1 // N16H14F1 // N10H21F1P1 // N12H18F1P2	1.9
51068.6	N10H21F2 // N12H18F2P1 // N14H15F2P2 // N14H16F2 // N10H20F2P2	1.1
51122.6	N11H20F1P2 // N9H22F1P3	1.0
51165.1	N10H22F1P1 // N12H19F1P2 // N14H17F1P1 // N12H20F1	2.9
51232.6	N14H17F2 // N10H22F2 // N12H19F2P1 // N14H16F2P2 // N9H20F4P3	2.7
51286.6	N13H19F1P1 // N11H21F1P2	1.0
51311.4	N10H22F2P1 // N12H19F2P2 // N14H17F2P1 // N12H20F2	0.2
51377.5	N12H19F3P1 // N14H17F3	0.3
51431.7	N11H21F2P2 // N13H19F2P1	0.7
51598.7	N15H18F2	0.9
